# Assessment of Butchers' Awareness, Attitude, and Occupational Practices Toward Crimean‐Congo Hemorrhagic Fever (CCHF) in Kabul, Afghanistan: A Cross‐Sectional Study

**DOI:** 10.1002/hsr2.71987

**Published:** 2026-03-02

**Authors:** Fazel Ahmad Muhammadi, Sayed Abdul Wahab Sadat, Rohullah Sakhi, Abdul Qaher Jasoor, Aimal Mohammadi, Niaz Mohammad Azizi, Hassan Hassanpoor, Abdulhanan Hanafi, Nader Qambari, Abdul Qayoom Joyenda, Mohammad Hussain Joya

**Affiliations:** ^1^ Kabul University of Medical Sciences, Public Health Faculty Kabul Afghanistan

**Keywords:** Afghanistan, attitude, awareness, CCHF, practice

## Abstract

**Background and Aims:**

Crimean‐Congo hemorrhagic fever (CCHF) is a viral disease with a fatality rate up to 40%, transmitted through tick bites and infected livestock. Afghanistan's livestock farming and limited public health infrastructure increase infection risks. This study assesses butchers' awareness, attitudes, and practices regarding CCHF in Kabul to identify gaps and inform targeted interventions.

**Methods:**

A cross‐sectional study was conducted among butchers in Kabul from August to September 2024 using cluster sampling. Data from 179 butchers across four districts were collected via questionnaires and analyzed with SPSS version 27.

**Results:**

The response rate exceeded 95%. Participants averaged 37 ± 13 years, with 31.8% having over 20 years of experience. Mean scores were 27.7 ± 3.8/38 for awareness, 18.4 ± 2.3/25 for attitudes, and 10.9 ± 2.3/16 for practices. While 41.9% showed good awareness and 61.5% had positive attitudes, only 42.5% demonstrated good practices. Significant misconceptions included belief in waterborne (52.5%) and airborne (31.8%) transmission. Unsafe practices like holding knives in the mouth (4.5%) and inconsistent protective equipment use were observed. A moderate positive correlation existed between attitudes and practices (r = 0.433, *p* < 0.001).

**Conclusion:**

A substantial gap exists between butchers' knowledge and practices regarding CCHF. Targeted educational interventions addressing attitudes and practical barriers are essential to improve safety practices.

## 1 Introduction

Crimean‐congo hemorrhagic fever (CCHF) is a significant zoonotic viral infection transmitted through tick bites and is recognized as one of the most widespread arboviral diseases globally [1]. The disease was first identified during an epidemic among Soviet troops in the Crimea in 1944 and was later isolated in the Congo in 1956, which established its transcontinental significance and provided its name [2]. In Afghanistan and across neighboring countries, CCHF represents a persistent and severe public health challenge. Outbreaks have been recurrent, with Pakistan confirming over 350 cases between 2014 and 2020—a sharp increase from its first case in 1976—and mortality rates exceeding 25% [3]. Similarly, China reported an initial outbreak in 1965 that led to 260 cases among farmers by 1994, with a mortality rate of 80% during that period. India documented its first cases in Gujarat and experienced 34 outbreaks from 2010 to 2019, while Iran observed its first human case in 1999, with mortality rates declining from 20% in 2000% to 6% by 2007 [3].

Within Afghanistan, the CCHF burden has risen sharply in recent years, a trend hindered by critical systemic limitations. The country faces constraints in laboratory testing capabilities and a lack of comprehensive clinical management knowledge, which obstructs timely diagnosis and effective treatment essential for outbreak control [4]. Epidemiological data from 2007 to 2018 confirm this increasing threat, recording 1,284 CCHF cases with a case fatality ratio of 43.3% [5]. The disease exhibits a distinct occupational pattern, disproportionately affecting butchers (6%), housewives (15%), healthcare workers (13%), and shepherds (11%). Butchers, in particular, face heightened risks due to direct exposure from handling raw meat and sharp tools [5]. This pattern is further corroborated by national reports, including 237 confirmed cases in 2017 [6]. Another systematic review covering 2010–2019 that identified 1,537 suspected cases, with the highest incidence and mortality in the western region. This review noted that cases predominantly involved males (2:1 ratio) aged 16–84 working in animal husbandry, agriculture, and healthcare, and it underscored an urgent need for improved control strategies [7]. Compounding the crisis, most patients seek hospital care only at advanced stages of infection, which diminishes treatment effectiveness and worsens health outcomes [8].

The effectiveness of prevention, therefore, critically depends on the knowledge, attitudes, and practices of high‐risk occupational groups. Previous studies reveal a concerning disparity between awareness and safe behavior, particularly among butchers. A study in Kabul City found that while 92% of butchers had heard of CCHF and 67.4% possessed good knowledge, high‐risk practices—including exposure to blood splashes on the face (45%), sustaining knife cuts (70.1%), and consuming raw liver (27.3%)—remained prevalent. Alarmingly, 37.9% of CCHF cases among butchers and their families were fatal, highlighting a dangerous gap between knowledge and practice [4]. This pattern is not isolated but is a consistent regional finding. Studies in Pakistan show that 78% of butchers are aware of CCHF, yet widespread misconceptions about transmission persist [9]. In Iran, 85% awareness contrasts with only 40% correctly identifying tick bites as a key transmission route [10]. Further studies from Turkey, India, Kenya, Rawalpindi, and Uganda document that acknowledgment of risk and protective measures rarely translates into consistent practice, with low utilization of personal protective equipment and persistence of hazardous behaviors [11, 12, 13, 14, 15]. Consequently, these studies collectively indicate a significant increase in CCHF cases among high‐risk populations with concurrently limited and often ineffective disease awareness. In Afghanistan, where CCHF incidence continues to rise, significant and specific gaps persist regarding accurate transmission knowledge and preventive practices. This study, therefore, aims to assess butchers' awareness, attitudes, and occupational practices toward Crimean‐Congo Hemorrhagic Fever (CCHF) in Afghanistan. By identifying the precise high‐risk behaviors in this vulnerable group, the research will generate evidence‐based recommendations to enhance public awareness and mitigate exposure risks.

### 1.1 Research Objective

The study objective is to assess the butcher's Awareness, attitudes, and occupational practices toward Crimean‐Congo hemorrhagic fever (CCHF) in Kabul, Afghanistan.

### 1.2 Research Questions

What are the levels of awareness, attitudes, and practices of butchers concerning Crimean‐Congo Hemorrhagic Fever (CCHF) in Kabul, Afghanistan?

### 1.3 Study Design and Setting

This study employs a questionnaire‐based cross‐sectional design and was carried out in Kabul City, Afghanistan's capital, from August to September 2024. Kabul City is divided into 22 districts, 16 of which are situated in the central area. Based on data from the Afghanistan Craftsmen and Shopkeepers Chamber, there are around 1,000 registered butchers spread across the 22 districts of Kabul City.

### 1.4 Sample Size Calculation and Sampling Strategy

This study employed a cluster sampling method to select participants from the 22 districts of Kabul City. To ensure representativeness and logistical feasibility, four districts (the 3rd, 10th, 13th, and 17th) were randomly selected. With the help of local district authorities, a complete list of all active butcher shops in these four districts was compiled, which identified a total of 188 butchers. Given the manageable total population size within the selected clusters and to achieve maximum representation, a census approach was deemed appropriate. Therefore, all 188 butchers were targeted for inclusion in the study. Data was collected through face‐to‐face interviews and questionnaires.

### 1.5 Data Collection and Instrument Development

Data were collected through structured face‐to‐face interviews, utilizing a pre‐designed questionnaire developed by researchers in Iran [10]. The questionnaire was adapted to align with the objective of the study, and a pilot test was conducted with 20 non‐surveyed participants, who were shopkeepers from nearby areas, to confirm that all questions were clear and comprehensible.

The questionnaire has four main parts. The first part gathered demographic details about the butchers, including their age, marital status, education level, work experience, and any history of infection. The second part evaluated awareness through 19 questions, with three possible responses: ‘Yes’, ‘No’, and ‘I don't know’. The scoring system was adapted from the original validated questionnaire [10]. Points were assigned as follows: 2 points for a correct answer, 1 point for ‘I don't know’, and 0 points for an incorrect answer. This scoring scale was chosen to distinguish between incorrect knowledge (a wrong belief) and a lack of knowledge (absence of information), a distinction important for the design of targeted health interventions. For reverse‐scored questions (items 3 to 7), this scoring scheme was applied inversely. The total possible score for the awareness section ranged from 0 to 38. The third part measured participants' attitudes using five questions, each offering five response options: completely agree, agree, neutral, disagree, and completely disagree. Responses were scored on a scale from 5 to 1, with reverse‐scored questions (questions 1 and 3) scored from 1 to 5. The scoring range in this section was between 0 and 25. Finally, the fourth part examined occupational practices through eight questions, with three response options: always, sometimes, and never. Scores ranged from 2 to 0, with reverse‐scored questions (questions 1, 2, and 4) adjusted from 0 to 2 points. The total possible scores in this section fell between 0 and 16.

In this study, based on research conducted in Rafsanjan, Iran [10], participants' levels of awareness, attitude, and occupational practices were categorized into three groups: those individuals scoring above 75% were labeled as “Good,” individuals scoring between 50% and 75% were classified as “Immediate,” and those scoring below 50% were categorized as “Poor.”

### 1.6 Data Analysis

Data were analyzed using IBM SPSS Statistics for Windows, Version 27. Descriptive statistics are presented as means and standard deviations (SD) for normally distributed continuous data, and as frequencies (n) and percentages (%) for categorical variables. The Pearson Chi‐Square test was used to assess associations between categorical variables (awareness, attitude, practice levels). The strength of linear relationships between total scores was measured using Pearson's correlation coefficient (r). A priori statistical significance was defined as a two‐sided *p*‐value of less than 0.05 (*p* < 0.05). *p*‐values less than 0.001 are reported as *p* < 0.001. This study was reported in accordance with the Strengthening the Reporting of Observational Studies in Epidemiology (STROBE) guidelines for cross‐sectional studies.

### 1.7 Ethics Considerations

The research was carried out in compliance with the principles of the Kabul University of Medical Science (KUMS) Research Directorate, and Ethical clearance was granted by the Ethical Review Board Committee of KUMS (KUMS‐ERB‐036) as it was an accomplishment of a bachelor's degree that the supervisor strictly followed. Before their involvement in the study, all participants provided written informed consent for both participation and publication. Participation in the research was voluntary, and strict confidentiality measures were implemented to safeguard participant information. The anonymity of data collection was maintained to uphold confidentiality standards.

## 2 Results

### 2.1 Participants' Demographic Profile

Of 188 butchers surveyed across four districts of Kabul, 179 participated (response rate > 95%). Participants had a mean age of 37 ± 13 years, with 31.8% (57/179) having over 20 years of experience. Key demographic and baseline characteristics are detailed in Table 1. Notably, 38.5% (69/179) were illiterate, and 37.4% (67/179) reported that a family member or friend had a history of CCHF infection, indicating potential community‐level exposure.

**Table 1:** Participant demographic characteristics (*N* = 179).

Variables	Frequency	Percentage
**Age**		
< 20	19	10.6
20–30	51	28.5
31–50	86	48.0
> 50	23	12.8
**Marital status**		
Single	31	17.3
Married	148	82.7
**Education**		
Illiterate	69	38.5
Primary school	66	36.9
High school graduate or above	44	24.6
**Economic status**		
Poor	72	40.2
Intermediate	94	52.5
Good	13	7.3
**Years of work experience**		
≤ 5	37	20.7
6–10	33	18.4
11–20	52	29.1
> 20	57	31.8
**Have you heard about Crimean‐Congo hemorrhagic fever?**		
Yes	168	93.9
No	11	6.1
**History of CCHF infection**		
Yes	67	37.4
No	112	62.6

*Note:* Data presented as *n* (%) where n represents the number of participants and the denominator is the total sample size (*N* = 179).

### 2.2 Overall Participants' Awareness, Attitude, and Practice

Overall, the participants achieved an average awareness score of 27.7 ± 3.8 out of 38, an average attitude score of 18.4 ± 2.3 out of 25, and an average practice score of 10.9 ± 2.3 out of 16.

As shown in Tables 2, 49.1% (75/179) had good awareness, and 61.5% (110/179) demonstrated positive attitudes. However, only 42.5% (76/179) exhibited good occupational practices, revealing a critical knowledge‐ and attitude‐to‐practice gap.

**Table 2:** Participant awareness, attitude, and practice (*N* = 179).

Variables	Frequency	Percentage
**Awareness**		
Good	75	41.9
Intermediate	100	55.9
Poor	4	2.2
**Attitude**		
Positive	110	61.5
Intermediate	69	38.5
**Occupational practices**		
Good	76	42.5
Intermediate	80	44.7
Poor	23	12.8

*Note:* Data presented as *n* (%). *N* = 179. Awareness, attitude, and practice levels were categorized as: Good (> 75%), Immediate (50%–75%), and Poor (< 50%) based on total possible scores.

### 2.3 Participants' Misconceptions in Awareness

Despite high overall awareness, specific and dangerous misconceptions were prevalent (Table 3). A majority, 52.5% (94/179), incorrectly believed CCHF is waterborne, and nearly one‐third, 31.8% (57/179), thought it was airborne, fundamentally misunderstanding its zoonotic, tick‐borne nature. This highlights a primary target for public health messaging: correcting core transmission beliefs.

**Table 3:** Frequency distribution of participants' responses to awareness questions regarding CCHF.

Awareness questions	Yes	No	I don't know
Is Crimean‐Congo Hemorrhagic Fever (CCHF) fatal?	165 (92.2%)	7 (3.9%)	7 (3.9%)
Are you aware that it is necessary to consult a doctor upon noticing suspicious symptoms of the disease?	173 (96.6%)	3 (1.1%)	3 (1.1%)
When observing ticks on livestock during slaughter, is removing or crushing them with hands correct?	40 (22.3%)	128 (71.5%)	11 (6.1%)
Does holding a knife in the mouth during slaughter increase the risk of transmitting Crimean‐Congo Hemorrhagic Fever?	158 (88.3%)	15 (8.4%)	6 (3.4%)
Is Crimean‐Congo Hemorrhagic Fever transmitted through contaminated water?	94 (52.5%)	35 (19.6%)	50 (27.9%)
Is Crimean‐Congo Hemorrhagic Fever transmitted through contaminated air?	57 (31.8%)	64 (35.8%)	58 (32.4%)
Is Crimean‐Congo Hemorrhagic Fever transmitted through milk?	109 (60.9%)	29 (16.2%)	41 (22.9%)
Is Crimean‐Congo Hemorrhagic Fever transmitted through tick bites?	152 (84.9%)	18 (10.1%)	9 (5.0%)
Is Crimean‐Congo Hemorrhagic Fever transmitted through blood?	152 (84.9%)	15 (8.4%)	12 (6.7%)
Is bleeding from body parts a symptom of livestock infected with Crimean‐Congo Hemorrhagic Fever?	93 (52.0%)	52 (29.1%)	34 (19.0%)
Is subcutaneous bleeding a symptom of Crimean‐Congo Hemorrhagic Fever?	76 (42.5%)	67 (37.4%)	36 (20.1%)
Is high fever a symptom of livestock infected with Crimean‐Congo Hemorrhagic Fever?	97 (54.2%)	53 (29.6%)	29 (16.2%)
Is fever a symptom of Crimean‐Congo Hemorrhagic Fever in humans?	85 (47.5%)	45 (25.1%)	49 (27.4%)
Is shivering a symptom of Crimean‐Congo Hemorrhagic Fever?	103 (57.5%)	50 (27.9%)	26 (14.5%)
Is bone pain a symptom of Crimean‐Congo Hemorrhagic Fever?	75 (41.9%)	46 (25.7%)	58 (32.4%)
Are aggressive behavior and restlessness symptoms of livestock infected with Crimean‐Congo Hemorrhagic Fever?	62 (34.6%)	68 (38.0%)	49 (27.4%)
Is it mandatory to wash clothes contaminated with blood and internal organs after slaughtering livestock?	170 (95.0%)	5 (2.8%)	4 (2.2%)
Is it essential to use gloves and appropriate protective clothing that covers the entire body during the slaughtering process?	175 (97.8%)	1 (0.6%)	3 (1.7%)
Can livestock infected with Crimean‐Congo Hemorrhagic Fever remain asymptomatic?	79 (44.1%)	59 (33.0%)	41 (22.9%)

*Note:* Data presented as *n* (%). *N* = 179 for all questions.

### 2.4 Participants' Attitudes

Table 4 demonstrates the frequency distribution of research participants' attitudes toward Crimean‐Congo Hemorrhagic Fever (CCHF). The findings reveal that 9.5% (17/179) of participants held incorrect attitudes regarding the removal of ticks during animal slaughter, which indicates incorrect or negative attitudes about the role of ticks in transmitting CCHF. Similarly, 26.3% (47/179) of participants completely agree that the consumption of fresh meat without proper preparation and inspection can pose significant health risks due to potential contamination. These findings emphasize the need for targeted educational interventions to correct attitudes about safe slaughtering practices.

**Table 4:** Frequency distribution of participants' responses to attitude questions regarding CCHF.

Attitude	Completely agree	Agree	I don't have any idea	Disagree	Completely disagree
If ticks are observed on the body of the animal during slaughter, they can be crushed by hand	17 (9.5%)	19 (10.6%)	1 (0.6%)	85 (47.5%)	57 (31.8%)
The person who carries out the slaughter of the animal must receive training in proper, hygienic, and correct slaughtering techniques before the slaughter	90 (50.3%)	75 (41.9%)	5 (2.8%)	8 (4.5%)	1 (0.6%)
Immediately after the slaughter of the animal, fresh meat and meat products can be used	47 (26.3%)	82 (45.8%)	11 (6.1%)	32 (17.9%)	7 (3.9%)
Individuals who slaughter animals must wear gloves and appropriate protective clothing that covers the entire body	93 (52.0%)	82 (45.8%)	4 (2.2%)	0	0
Those who carry out the slaughter of animals must wash their blood‐stained clothes and the remains of the animal after slaughter	89 (49.7%)	86 (48.0%)	3 (1.7%)	1 (0.6%)	0

*Note:* Data presented as *n* (%). *N* = 179 for all questions.

### 2.5 Participants' High‐Risk Occupational Practices

Table 5 presents the findings of a study concerning animal slaughter practices and adherence to safety and hygiene standards. The results highlight the presence of unsafe behaviors in certain cases. Regarding tick bites, 8.9% (16/179) of respondents reported being consistently bitten by ticks, while 27.4% (49/179) experienced this occasionally. This indicates a significant potential risk for the transmission of zoonotic diseases, such as Crimean‐Congo hemorrhagic fever. Furthermore, 4.5% (8/179) of participants admitted to holding the knife in their mouth during slaughter, a highly unhygienic and hazardous practice that could lead to contamination. Additionally, only 20.1% (36/179) consistently used protective eyewear, which increases the likelihood of exposure to blood and other contaminated fluids. These findings emphasize the importance of education and the promotion of safe practices to minimize health risks and prevent the spread of infectious diseases.

**Table 5:** Frequency distribution of participants' responses to practice questions regarding CCHF.

Practice	Always	Sometimes	Never
Have you ever been bitten by a tick?	16 (8.9%)	49 (27.4%)	114 (63.7%)
Do you hold your knife in your mouth while slaughtering?	8 (4.5%)	12 (6.7%)	159 (88.8%)
Do you wear boots when slaughtering animals?	141 (78.8%)	22 (12.3%)	16 (8.9%)
Have you ever been contaminated with blood, organs, and entrails while slaughtering animals?	98 (54.7%)	50 (27.9%)	31 (17.3%)
Do you wear appropriate long clothing that covers your entire body when slaughtering animals?	134 (74.9%)	27 (15.1%)	18 (10.1%)
Do you wear a mask when slaughtering animals?	124 (69.3%)	26 (14.5%)	29 (16.2%)
Do you use gloves when slaughtering animals?	125 (69.8%)	29 (16.2%)	25 (14.0%)
Do you use safety glasses when slaughtering animals?	36 (20.1%)	18 (10.1%)	125 (69.8%)

*Note:* Data presented as *n* (%). *N* = 179 for all questions.

### 2.6 Relationship Between Awareness, Attitude, and Practice

The Pearson Chi‐Square test was used to evaluate the relationship between categorical variables (awareness, attitude, and practice). The results indicated a significant association between attitude and occupational practices (*p*< 0.001), but no association was found between awareness and practice (Table 6).

**Table 6:** Pearson Chi‐Square Test result.

Variables	Performance			Chi‐square *p* value
	Good	Immediate	Poor	
**Awareness**				
Good	32	37	6	*p* = 0.810
Intermediate	42	43	15	
Poor	2	0	2	
**Attitude**				
Good	60	42	8	*p* < 0.001
Intermediate	16	38	15	

*Note:* Data presented as frequency count (*n*). *p*‐value from the Pearson Chi‐Square test.

To assess the strength and direction of this relationship, correlation analysis was applied. The analysis revealed a moderate positive correlation between attitude and occupational practices (r = 0.433, *p* < 0.001). This suggests that higher attitude scores are associated with higher practice scores. In contrast, the correlation between practices and awareness was weak and not statistically significant.

## 3 Discussion

This study assessed awareness, attitudes, and occupational practices regarding CCHF among butchers in Kabul, Afghanistan. The findings reveal a significant disparity between theoretical knowledge and practical implementation of safety measures, highlighting the need for targeted interventions to reduce CCHF transmission risks.

The study demonstrated that a significant proportion of butchers (93.9%) had heard about CCHF, with 41.9% showing good awareness and 61.5% displaying positive attitudes. However, only 42.5% exhibited good practices, indicating a disconnect between knowledge and action. This discrepancy is consistent with previous studies in other regions, such as Pakistan and Iran, where high awareness levels did not always translate into safe practices [9, 10]. The recent distribution pattern analysis of CCHF in Asia and the Middle East further confirms that environmental and occupational exposure factors contribute significantly to this gap [16]. Most respondents strongly believe that the disease is fatal, which is in line with the Uganda study [15]. This alignment may be due to the disease's high fatality rate in both countries, as also observed in recent case studies from Iraq [17]. Misconceptions about transmission routes, such as contaminated water (52.5%) and air (31.8%), were prevalent, underscoring the need for precise educational campaigns to address these gaps. A similar study conducted in Kabul city also highlighted risky behaviors and knowledge gaps among butchers, reinforcing the need for localized interventions [4]. The study also identified unsafe practices among butchers, such as holding knives in their mouths during slaughter (4.5%), and the inconsistent use of protective equipment. These findings align with research from India and Kenya, where similar risky behaviors were reported [12, 13]. The demographic profile of participants revealed that 38.5% were illiterate, which may contribute to the gap between awareness and practices. This finding is consistent with studies in Afghanistan and Pakistan, where low literacy levels were associated with poor adherence to safety measures [6, 9]. The epidemiology of CCHF in Afghanistan, as reported through national surveillance, indicates that occupational exposure remains a key risk factor, supporting the need for targeted interventions [5]. A deeper analysis of the barriers reveals that low literacy constrains the understanding of written guidelines, entrenched occupational habits resist simple awareness, and economic limitations hinder access to protective equipment. Therefore, interventions must be practical and multifaceted. Educational campaigns should use visual and oral methods tailored for low‐literacy learners, focusing on correcting specific high‐risk misconceptions and practices. Occupational guidance should promote enforceable, low‐cost safety protocols in slaughterhouses, such as mandatory glove use and supervised training in tick removal and tool hygiene. The study revealed a moderate overall knowledge score (27.7 ± 3.8/38, 73%), with 93% of butchers having heard of CCHF, consistent with reports from endemic regions like Pakistan [9]. However, significant misconceptions persisted, including beliefs in waterborne (52.5%) and airborne (31.8%) transmission, echoing findings from Iran, where butchers misunderstood tick transmission roles [10]. These knowledge gaps, particularly regarding symptoms like subcutaneous bleeding (unknown to 37.4%) and asymptomatic livestock (unknown to 33%), align with studies in Turkey and reflect persistent challenges in Afghanistan [7, 11]. While attitudes were generally positive (61.5%), negative attitudes toward tick removal (9.5%) highlighted critical prevention barriers, consistent with findings from Nangarhar, Afghanistan [18]. Most notably, a stark knowledge‐practice gap was observed, with only 42.5% demonstrating good practices. Unsafe behaviors, such as tick bites (8.9% consistently, 27.4% occasionally), knife‐in‐mouth use (4.5%), and low protective eyewear adherence (20.1%), were consistent with reports from Mauritania, Nigeria, and Afghan clinical studies [8, 19, 20].

Statistical analysis confirmed a moderate positive correlation between attitude and practice (r = 0.433, *p* < 0.0001), while awareness alone showed no significant link to practice. This aligns with the Health Belief Model and studies from India, emphasizing that knowledge must be coupled with perceived risk and practical solutions to drive behavioral change [21, 22]. Underlying factors such as illiteracy (38.5%), economic barriers, and entrenched habits (31.8% with > 20 years' experience) further hindered compliance, as supported by zoonotic disease research [23]. These findings underscore the need for targeted, practical interventions addressing attitudes and contextual barriers rather than knowledge dissemination alone. This study is subject to certain limitations. Its cross‐sectional design precludes the determination of causal relationships between variables. Additionally, the study was conducted in Kabul, and the findings may not be fully generalizable to butchers in rural areas of Afghanistan or to other high‐risk occupational groups, which may face different contextual challenges.

## 4 Conclusion and Recommendation

In conclusion, this study identifies a critical gap between the high awareness and the inadequate safety practices of butchers in Kabul regarding CCHF. To bridge this gap and mitigate transmission risk, a multi‐level approach is required. Future research should include longitudinal studies to establish causality between attitudes and behavior, and operational research to test low‐cost interventions in Afghanistan. Intervention strategies must shift from generic awareness to tailored, visual, and practical education—correcting key misconceptions and demonstrating skills like safe tick removal. Policy measures should enforce low‐cost safety protocols, such as mandatory protective equipment use in slaughterhouses, supported by potential subsidies. Integrating targeted education, practical training, and supportive policy can effectively translate knowledge into safer practices, reducing the occupational risk of CCHF in Afghanistan.

## Author Contributions

**Fazel Ahmad Muhammadi:** conceptualization, investigation, methodology, validation, visualization, writing – review and editing, software, formal analysis, project administration, data curation, supervision, resources, writing – original draft. **Sayed Abdul Wahab Sadat:** investigation, methodology, validation, writing – review and editing, software, formal analysis, project administration, data curation, supervision, resources, conceptualization, visualization. **Rohullah Sakhi:** investigation, methodology, validation, visualization, writing – review and editing, software, formal analysis, project administration, data curation, supervision, resources, conceptualization. **Abdul Qaher Jasoor:** investigation, data curation. **Aimal Mohammadi:** investigation, data curation. **Niaz Mohammad Azizi:** investigation, data curation. **Hassan Hassanpoor:** investigation, data curation. **Abdulhanan Hanafi:** investigation, data curation. **Nader Qambari:** investigation, data curation. **Abdul Qayoom Joyenda:** investigation, data curation. **Mohammad Hussain Joya:** investigation, data curation.

## Funding

The authors received no specific funding for this work.

## Ethics Statement

This study was approved by the Ethical Review Board Committee of Kabul University of Medical Sciences (KUMS) (Approval No: KUMS‐ERB‐036). Written informed consent was obtained from all individual participants included in the study.

## Conflicts of Interest

The authors declare no conflicts of interest.

## Transparency Statement

The lead author Fazel Ahmad Muhammadi affirms that this manuscript is an honest, accurate, and transparent account of the study being reported; that no important aspects of the study have been omitted; and that any discrepancies from the study as planned (and, if relevant, registered) have been explained.

## Data Availability

The data that support the findings of this study are available from the corresponding author, Fazel Ahmad Muhammadi, upon reasonable request.
